# Conditional Cell Reprogramming in Modeling Digestive System Diseases

**DOI:** 10.3389/fcell.2021.669756

**Published:** 2021-06-03

**Authors:** Ruihua Zhao, Rui Li, Tianqi An, Xuefeng Liu

**Affiliations:** ^1^Department of Oncology, The First Affiliated Hospital of Zhengzhou University, Zhengzhou, China; ^2^Department of Oncology, Tongji Hospital, Huazhong University of Science and Technology, Wuhan, China; ^3^Department of Pathology, Center for Cell Reprogramming, Georgetown University Medical Center, Washington, DC, United States; ^4^Department of Oncology, Lombardi Comprehensive Cancer Center, Georgetown University Medical Center, Washington, DC, United States; ^5^Departments of Pathology and Urology, The Ohio State University School of Medicine, Columbus, OH, United States; ^6^James Comprehensive Cancer Center, The Ohio State University, Columbus, OH, United States

**Keywords:** conditional cell reprogramming, digestive system diseases, cell model, CR technology, cell culture technology

## Abstract

Digestive diseases have become an important source of morbidity and mortality. The considerable financial and health burdens caused by digestive diseases confirm the importance of extensive research to better understand and treat these diseases. The development of reliable preclinical models is essential for understanding the pathogenesis of digestive diseases and developing treatment and prevention methods. However, traditional established cell lines and animal models still have many limitations in the study of the digestive system. Conditional reprogramming (CR) cell culture is a newly developed primary technology that uses irradiated Swiss-3T3-J2 mouse fibroblast cells and the Rho-associated kinase (ROCK) inhibitor Y-27632 to rapidly and efficiently generate many cells from diseased and normal tissues. CR cells (CRCs) can be reprogrammed to maintain a highly proliferative state and recapitulate the histological and genomic features of the original tissue. Moreover, after removing these conditions, the phenotype was completely reversible. Therefore, CR technology may represent an ideal model to study digestive system diseases, to test drug sensitivity, to perform gene profile analysis, and to undertake xenograft research and regenerative medicine. Indeed, together with organoid cultures, CR technology has been recognized as one of the key new technologies by NIH precision oncology and also used for NCI human cancer model initiatives (HCMI) program with ATCC. In this article, we review studies that use CR technology to conduct research on diseases of the digestive system.

## Introduction

The digestive system involves the gastrointestinal tract, including the oral cavity, pharynx, esophagus, stomach, small and large intestines, as well as auxiliary organs, namely, the liver, gallbladder, and pancreas. The digestive system is a continuous anatomical structure that can swallow, digest, absorb food nutrients, and expel the remaining waste. It also mediates interactions between the host and resident bacteria (Matuchansky and Beauchant, 1982). Digestive system diseases (including infections, inflammation, and cancers) have become important sources of morbidity and mortality. Globally, the burden of digestive system diseases is increasing annually (GBD 2017 Pancreatic Cancer Collaborators, 2019; GBD 2017 Inflammatory Bowel Disease Collaborators, 2020; GBD 2017 Oesophageal Cancer Collaborators, 2020). Colorectal and stomach cancers are the third and fourth most common malignancies worldwide, each accounting for approximately 800,000 victims per year (Siegel and Miller, 2020). Other common benign digestive system diseases, such as acute and chronic gastroenteritis caused by bacteria or other pathogens, inflammatory bowel disease (IBD), and functional gastrointestinal disorders, not only cause pain, disability and even death but also carry an enormous financial burden to both families and society. Indeed, the direct costs for care of patients with IBD singly were estimated to be €4.6–5.6 billion annually in Europe, and the incidence of IBD is still increasing steadily in Western countries (Burisch et al., 2013). The enormous financial and health burdens resulting from digestive system diseases confirm the importance of great research efforts to better understand and treat these diseases.

Developing reliable preclinical models that can accurately recapitulate the complex physiology and pathophysiology of different digestive system organs is essential to understand the developmental processes and mechanisms of diverse disorders of the digestive system organs, such as infection, inflammation, and cancer, to develop drug therapy. Conventionally, traditional established cell lines cultured in 2-dimensional (2D) or animal models have been widely used as digestive system models. Although these models have significantly contributed to the development of digestive system research, they still have many limitations. Only 1–10% of cell lines (depending on the source tissue and disease progression status) can be successfully established to be transformed immortalized or cancerous cell lines, and most primary cells, especially normal cells, are hard to culture because of their limited life span (Giard et al., 1973). More importantly, after being artificially cultured on glass or plastic plates with undefined medium for the long term, the genes of the established cell lines have undergone tremendous changes, making it impossible to recapitulate the complex characteristics of the primary tissue. Therefore, the results obtained in 2D cultures may not represent the true host response (Gillet et al., 2013). Animal models, especially recently emerged engineered mouse models and primary patient-derived xenografts (PDXs), may overcome the limitations of cell lines and better mimic human disease and treatment response (Ito et al., 2018). However, the application of animal models might be limited by high costs, low throughput, and technical challenges; more importantly, differences in specific species cause inaccurate recapitulation of biological and therapeutic responses. Recently, *in vitro* three-dimensional (3D) organoid culture techniques for various cell types, such as induced pluripotent stem cells (iPSCs), pluripotent embryonic stem (ES) cells, and immortalized cell lines, have been successfully developed (Kretzschmar and Clevers, 2016; Sato et al., 2009). Three-dimensional organoid models contain multicellular organ structures, which are thought to closely mimic complex original structures and functions. They can also be maintained for a long time and are easily manipulated (Clevers, 2016). Digestive system organoids have been established using cells from the stomach, small intestine, colon, and other organs (Pan et al., 2018). Organoids have advantages in understanding the mechanisms and biological processes of digestive diseases (such as cancer, infectious disease, and IBD), thereby helping to promote the development of personalized and regenerative medicine. However, they are not suitable for high-throughput screening because in general, 28–42 days are needed to grow enough cells (Xinaris, 2019). There is still an urgent need for a single model of the digestive system that is fast, easy to execute, and easily successful.

**FIGURE 1:**
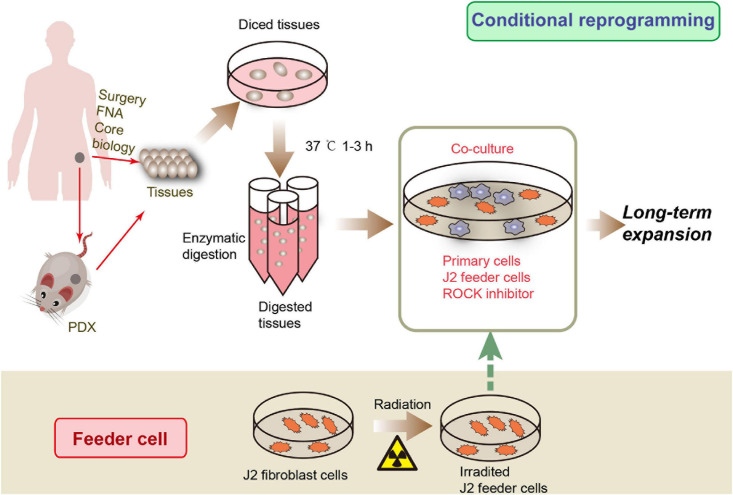
CRC development processes. Tissue samples can be obtained from surgical core biopsies, fine-needle aspiration (FNA) or patient-derived xenograft (PDX). The tissue is then cut into small pieces and digested to produce primary cells. Then the primary cells were co-cultured with irradiated J2 feeder cells and ROCK inhibitor to obtain CR cells.

Recently, Liu et al. (2017) developed a new primary cell culture technology, called conditional reprogramming (CR), using irradiated Swiss-3T3-J2 mouse fibroblast cells and Y-27632, a Rho-associated kinase (ROCK) inhibitor, to rapidly and efficiently generate indefinite epithelial cells (Figure 1). Cells processed by this method are called conditionally reprogrammed cells (CRCs). The CR method can rapidly and efficiently generate large numbers of primary epithelial cells from different tissues, such as fresh or cryopreserved surgical specimens, fine-needle aspiration (FNA), core biopsies, and PDX tissues (Palechor-Ceron et al., 2019). CRCs can be reprogrammed to maintain a highly proliferative state, known as “reprogrammed stem-like” (Suprynowicz et al., 2012), and recapitulate the histological characteristics and genomic characteristics of the original tissue (Alamri et al., 2016). Moreover, after removing these conditions, the phenotype is completely reversible (Liu et al., 2012, 2020). Therefore, CR technology might be an ideal model to study digestive system diseases, to test drug sensitivity, to perform gene profile analysis, and in xenograft research and regenerative medicine. In this article, we review studies that use CR technology for digestive system disease research (Table 1).

**TABLE 1:** Comparison of the model systems for digestive system diseases.

	Conventional cell lines	Primary cells	PDX model	3D organoid	CRC
**Sample origin**					
FNA	No	No	No	No∼+	+++
Core biopsy	No	No∼+	-	+	+++
Surgical specimens	+	++	++	+++	+++
Cryopreserved tissue	No∼+	+∼++	No∼+	+++	+++
Cancerous tissue	+++	++	++	+++	+++
Noncancerous tissue	No	No∼+	No	+	+
Timing	Several days	1∼4 weeks	1∼5 months	1∼4 weeks	1–10 days
Success rate	+	++	++	+++	+++
Rapid expansion	+++	++	+	++	+++
Genetic stability	+	++	++	++	++
Cost	+	++	+++	++	+
HT screening	+++	+	No	++	+++
Representation of primary tissue	+	++	++	++	++
Life span	+++	+	+	++	+++
Difficulty of differentiation	+++	+	+++	+	+
biobanking	No	+	++	+++	+++
Tissue-specific	+	+++	+++	+++	+++
Genetic manipulation	+++	No∼+	No	++	++
Tumor–stroma interaction	No	No	++	+	No

*This table was adapted and modified from Liu and Mondal (2020).*

## Development of CR Technology

The *in vitro* life spans of primary cells, including normal human epithelial cells and human embryonic stem cells (hESCs), are very short, which is an obstacle to research (Reubinoff et al., 2000). Different efforts have been made to optimize the cultivation of primary cells. Initially, H Green developed a keratinocyte/feeder coculture system. By using lethally irradiated feeder cells at the correct density, keratinocytes can be continuously propagated (Rheinwald and Green, 1975). The method was further developed by adding an epidermal growth factor (Stanley and Dahlenburg, 1984). Y-27632 was initially proven to significantly improve the cloning efficiency of human embryonic stem (ES) cells (Watanabe et al., 2007), and a study found that using Y-27632 during primary culture can effectively prepare large numbers of human epithelial stem cells from various primitive epithelial tissues (Terunuma et al., 2010). Sandra et al. also reported that treatment of primary keratinocytes with Y-27632 can greatly improve the proliferation and immortality of these cells (Chapman et al., 2010; Liu et al., 2012). More importantly, immortalized cells showed typical characteristics of primary keratinocytes. Later, Liu and colleagues proved that the combination of Y-27632 and fibroblast cells, called CR technology, can induce indefinite proliferation of many epithelial and nonkeratinocyte normal cells and tumor cells *in vivo* (Liu et al., 2012, 2017; Tricoli et al., 2018). The CR method has been shown to be rapid and efficient in the establishment of cell cultures capable of unlimited growth from normal and cancer tissues. Most importantly, CRCs maintain the developmental potential of the original tissue and can restore the differentiation ability of the cells after these conditions are removed. Additionally, the CR method can rapidly generate cultures from small biopsy specimens and cryopreserved tissue. Recently, the technology has been modified and can be used to cultivate cells from the skin, prostate, lung, breast, kidney, and even neuroendocrine and endocrine tissues (Gao et al., 2017; Liu et al., 2017; Palechor-Ceron et al., 2019; Timofeeva et al., 2017; Urbaniak et al., 2019). Therefore, the CR method has potential applications in basic research and diagnosis, treatment, and regenerative medicine.

## Application in Digestive Diseases Research

### Modeling Diseases

CRCs have been established quickly and efficiently from a variety of human normal and tumor specimens, including the colon. There is no need to use exogenous viruses or genetic manipulation. CRCs can maintain the characteristics of their primary tissues. After removing these conditions, the differentiation ability of the cells can be restored, and they can be grown in 2D and 3D systems, making CR an ideal *in vitro* model of digestive diseases. Emily C et al. applied CR technology to establish a 2D monolayer model of mouse intestinal epithelial cells (IEC monolayers) from both wild-type and genetic mouse models. IEC monolayers can form epithelial colonies and maintain their genetic characteristics during the long passage process. They can also form 3D spheres in medium, including Matrigel (Moorefield et al., 2018). This study indicates that CR technology could be used to culture numerous genotype-specific mouse small intestinal epithelial cells for further functional studies. Recently, using CR technology, Su et al. successfully isolated human primary hepatocytes from fresh liver tissues of patients of different ages and with different hepatic diseases. They achieved a high success rate in the long-term culture of primary hepatocytes *in vitro* with various hepatic diseases (Su et al., 2019b). They also established a primary human hepatocyte culture with liver function from patients with ornithine transcarbamylase deficiency (Su et al., 2019a). Therefore, the study showed that the use of CR technology in patient-derived hepatic cell culture can be a valuable and viable model for studying mechanisms related to hepatic diseases. Using CR technology, a group of well-differentiated porcine pancreatic ductal epithelial cells with bicarbonate secretion capacity have been established, which will improve our knowledge of pancreatic physiology and bicarbonate secretion mechanisms (O’Malley et al., 2018). For the establishment of preclinical cancer models, it is essential to obtain enough tumor tissues. However, tissue samples can only be obtained from many advanced cancer patients through fine-needle or core needle biopsy. Research by Lee et al. (2019, 2021) showed that CR technology can be used to establish pancreatic cancer cell lines through biopsies guided by endoscopic ultrasound. CRCs from pancreatic cancer tissues have the same genetic characteristics as those from the primary tumor and can be used for genomic research and drug sensitivity studies and can identify new diagnostic and therapeutic targets for pancreatic cancer (Lee et al., 2019, 2021). The PDX model has become an important tool for translational research, but there are some limitations, including unsustainable *in vitro* growth. Researchers used CR technology to successfully generate and amplify stable cell lines from PDX tumors of human bladder, lung, and ovaries without compromising the basic biological characteristics of the model (Borodovsky et al., 2017). Therefore, CR technology can be used for *in vitro* amplification of PDX cells for subsequent studies. CR cells can be produced from organoid and xenograft tissues and can also form CR cell-derived xenograft (CDX) tumors and be cultured in spheres or organoids (Timofeeva et al., 2017; Moorefield et al., 2018; Mondal et al., 2019; Palechor-Ceron et al., 2019), demonstrating that these three platforms can work together to provide platforms for digestive system disease study.

### Precision Medicine and Drug Discovery

Precision medicine is a newly developed method for the treatment and prevention of diseases based on the patients’ biological information and their clinical signs and symptoms (Collins and Varmus, 2015). Recently, in oncology, the treatment and classification of cancer have changed due to genetic testing. Targeted therapy is a treatment method that uses drugs to target specific genes and proteins related to the survival and growth of cancer cells, and this is the foundation of precision medicine. Targeted therapy is a rapidly developing field of cancer research. Researchers are studying many new targets and drugs through basic and clinical research (Baudino, 2015). Although the outcomes of certain cancer patients, including patients with lung and breast cancers, have been greatly improved due to targeted therapies (Gu et al., 2016; Hirsch et al., 2017), the results of evaluating new targeted drugs in digestive system cancer clinical trials are usually frustrating. In the past 20 years, targeted therapy for gastrointestinal cancer has made little progress (Narita and Muro, 2017; Aslan et al., 2018; DeLeon et al., 2018; Dzunic et al., 2019). A particular limitation in identifying new and effective targets and drugs for digestive system cancers is that the results are only based on studies in long-term cultured cell lines or xenograft models (Garnett et al., 2012); thus, the results of most clinical trials are usually disappointing. Currently, the use of patient-derived models with the advantages of stable genotype, high-throughput screening, immortality, and xenotransplantation is urgently needed for target discovery and drug screening. CR technology can be used in primary cell cultures from normal and tumor tissues of different species and to maintain the phenotypic and genotypic characteristics and intratumoural heterogeneity of the primary tissues. CR cells can also be cultured in PDX models and 3D conditions. Therefore, CR technology provides a new platform for evaluating the effectiveness and toxicity of new drugs and developing individualized treatment plans in digestive system diseases. Recently, Alamri et al. used CR technology to cultivate CRCs from primary tissue of low-grade mucoepidermoid carcinoma (MEC) and used them to detect candidate therapeutic pathways. They demonstrated that the amphiregulin-mechanistic target of rapamycin-protein kinase B (AKT; AKT1) pathway was activated in MECs and that the growth of MECs could be inhibited by MK2206 (allosteric AKT inhibitor) in 2D and 3D cultures (Alamri et al., 2018). The CR technique was used for the first time to determine the important role of the MYC-ERCC3 interaction in pancreatic ductal adenocarcinoma (PDAC), and triptolide (a covalent ERCC3 inhibitor) was found to be a potential treatment target in MYC-dependent PDAC (Beglyarova et al., 2016). Another group used PDAC cell lines and CR-cultured primary cells to identify the role of the low-immunogenicity anti-mesothelin immunotoxin RG7787 in pancreatic cancer (Hollevoet et al., 2014). Wang et al. established a novel individualized CR system (termed i-CR) from colorectal cancer tissues. By high-throughput i-CR drug screening, they discovered that inhibition of the EGFR and MEK or CDK4/6 pathways exerted a synergistic inhibitory effect against colorectal cancer and supersynergistic effects when EGFR, MEK, and CDK4/6 inhibitors were used simultaneously. Their study showed that the novel i-CR system combined with PDX models will enable individualization of therapy and drug discovery (Wang et al., 2020). Using CRCs from colorectal cancer patients, Kim et al. (2018) found that ATP6V0C encoding lysosomal V-ATPase V0 subunit C and IDF-11774 (a new clinical drug candidate for the treatment of colorectal cancer) are synthetically lethal and that this effect was associated with the expression of B-cell CLL/lymphoma 2 (Bcl-2) and PIK3CA mutations. Adenoid cystic carcinoma (ACC) is a rare salivary adenocarcinoma and has a high rate of metastasis. Currently, we still do not have a research model suitable for this disease (Bradley, 2017). Chen et al. (2017) successfully cultured ACC cells from two separate ACC PDX tumors using modified CR medium conditions (which can maintain the characteristic MYB translocation). They also developed a method to rapidly verify the ability of cultured CR cells to metastasize *in vivo* using zebrafish. Using the CR/zebrafish model, they identified regorafenib as a potential drug for this cancer. In another study using CR technology, Panaccione et al. (2016) found that ACC features previously unidentified CD133+ cells with neural stem characteristics. These cells were driven by FABP7, NOTCH1, and SOX10 expression. They also found that these cells were sensitive to Notch inhibition, which may provide a new target for the treatment of ACC. In addition, some groups have used patient-derived CRCs from other cancers for comprehensive drug testing (Saeed et al., 2017; Kettunen et al., 2019). A recently published article explored the clinical feasibility of using CRC to guide chemotherapy for patients with colorectal cancer. They generated CRC cells and paired them with a PDX mouse model of primary colorectal tumor cells to establish the correlation between drug sensitivity *in vitro* and the patient’s clinical response. They showed that the use of CRC screening chemical drug screening is comparable to the PDX model. More importantly, it was shown to be highly consistent with the clinical results of the enrolled colorectal cancer patients (Li et al., 2021). Therefore, we believe that this technology will be used for high-throughput drug screening of digestive system diseases, especially digestive system tumors. Su et al. (2019a) established CRCs from fresh human liver tissues and determined that the cells still reserve 1A1, 2C9, and CYP3A4 activities. CYPs are essential for drug metabolic enzymes in the human body. Therefore, CRCs from primary normal cells might be used as a model for evaluating drug toxicity and metabolism.

### Regenerative Medicine

Regenerative medicine is a science and technology that uses biological and engineering methods to create lost or functionally damaged tissues and organs to establish their normal function. The field mainly includes cell therapy, immunomodulatory therapy (separate administration or secretion by injection of cells and regeneration of bioactive molecules), and tissue engineering (transplantation of organs and tissues grown in the laboratory). This technology provides new treatments for patients suffering from clinical problems such as end-stage organ failure and serious injuries (Atala, 2012). In recent years, stem cells, including adult stem cells (ASCs), induced pluripotent stem cells (iPSCs), and embryonic stem cells (ESCs), have been studied intensely to explore their value in regenerative medicine (Riazi et al., 2009). However, there is some difficulty in efficiently inducing the functional differentiation of stem cells. CR technology can generate cells from very few donor tissues and can rapidly expand them *in vitro* in large quantities; more importantly, the cells can be well differentiated into the native cell type after CR conditions are eliminated. Therefore, CR technology has application potential in the field of regenerative medicine. Using CR-cultured cells for tissue engineering and regenerative medicine has aroused great interest among scientists. Cells can be cultured using CR technology and engineered methodologies, particularly 3D scaffold materials, to generate tissues that exquisitely recapitulate their original structures and functions. Another method of using CR technology for regeneration is to promote their maturation into functional tissues and then implant them in the host. Hamilton et al. implanted a combination of airway epithelial CR cells and a fibroblast-containing graft in a rabbit model of revascularization, featuring decellularized tracheal stents and implanted structures with the function of revascularization. They found keratin-positive cells throughout the scaffold (Hamilton et al., 2019). Their research showed that CR cells can improve the repair of host epithelium and/or directly promote mucosal regeneration, which can be used in regenerative medicine. One study showed that CR technology can indefinitely expand normal oesophageal epithelial cells from endoscopic biopsy samples of pediatric patients (Jensen et al., 2017). Therefore, the cells can be combined with synthetic or natural scaffolds to provide treatment options for patients with defects, trauma, or diseases. Having a large number of cells will help to construct a fully regenerated oesophageal epithelial cell cavity to help promote the regeneration of the remaining cell types. Therefore, rapid amplification of patient-derived cells with stable genetic characteristics based on CR technology will meet the unmet needs of tissue engineering for personalized regenerative medicine in digestive system disease. Further research in these subfields may lead to the development of treatment options to use different types of CR cells in regenerative medicine for human digestive system diseases.

## Others

Biobanks play vital roles in innovative clinical medicine and translational biomedical research. The latest scientific advances in cryopreservation have made it possible to establish living biobanks, which enable long-term collection and storage of viable functional tissues and proliferating cell types (Li et al., 2020). The ability to quickly and efficiently build a stable CRC from normal and diseased tissue provides an exciting opportunity to build a living biobank (Liu et al., 2012; Coppola et al., 2019). Our team proposed establishing the Next Generation Living Biobank (NGLB), which collects and stores tissues from surgery, core needle or fine-needle biopsy, and cells from scrub or liquid, such as urine and blood. Corresponding omics (e.g., genomics, transcriptomics, proteomics, metabolomics) and clinical information should be gathered together. At the same time, CR technology is being used to grow large numbers of cells to meet the needs of disease modeling, drug discovery, and regenerative medicine in the future (Palechor-Ceron et al., 2019). Compared with traditional living biobanks, NGLB enables us to use CR technology to generate cells in rare diseases for which cell lines or cell models are not currently available (Table 2).

**TABLE 2:** Applications of CR technology in digestive system diseases.

Origination	Tissue	Cells	Application	References
			**Cell model**	
Genetic mouse model	Intestinal	Normal intestinal epithelium	Genotype-specific mouse intestinal epithelial cells	Moorefield et al., 2018
Human	Liver	Hepatocytes	Hepatic cells from different liver diseases: OTCD; Citrullinemia type 1 disease; Cirrhosis; Hepatitis C virus-induced liver failure; Maple syrup urine disease	Su et al., 2019a,b
Sus scrofa	Pancreas	Pancreatic ductular epithelium cell	Pancreatic ductular epithelial cells	O’Malley et al., 2018
Human	Pancreas	Pancreatic cancer cell	Pancreatic cancer cells obtained by EUS-FNB.	Lee et al., 2019
			**Precision medicine and drug discovery**	
Human	Pancreas	Pancreatic ductal adenocarcinoma cell	Determine the important role of the MYC-ERCC3 interaction in PDAC, found triptolide may be a potential target treatment for MYC-dependent PDAC	Beglyarova et al., 2016
Human	Pancreas	Pancreatic ductal adenocarcinoma cell	Identify the role of the low immunogenicity anti-mesothelin immunotoxin RG7787 in pancreatic cancer	Hollevoet et al., 2014
Human	Salivary gland	Salivary gland neoplasms	Demonstrated AKT; the AKT1 pathway was activated in MEC, and the growth of MEC cells can be inhibited by MK2206	Alamri et al., 2018
Human	Large intestine	Colorectal cancer	Identify synergistic effects of EGFR, MEK, and CDK4/6 inhibitors in the colorectal cancer	Wang et al., 2020
Human	Large intestine	Colorectal cancer	Identified that ATP6V0C and IDF-11774 were synthetically lethal and this effect was associated with low Bcl-2 expression and PIK3CA mutations	Kim et al., 2018
Human	Large intestine	Colorectal cancer	For rapid screening of individualized chemotherapy for colorectal cancer patients	Wang et al., 2020
Human	Salivary gland	Adenoid cystic carcinoma	Identified regorafenib as a potential therapeutic drug	Chen et al., 2017
Human	Salivary gland	Adenoid cystic carcinoma	Characterized the majority population of CD133+ cells in ACC and found these cells were sensitive to Notch inhibition	Panaccione et al., 2016
			**Regenerative medicine**	
Human	Oesophageal	Oesophageal epithelial cells	Might help to engineer an oesophageal construct with a completely reseeded oesophageal epithelial cell lumen	Riazi et al., 2009

### Limitations of CR Technology

The results of CR technology studies have proven that it may play an important role in digestive system disease. However, some limitations need improvement. The CR method cannot lack stromal components such as matrix, vascular immune, or endothelial cells. Therefore, we were unable to analyze the effect of stromal cells on tumor cell growth and their response to therapeutic agents (Liu et al., 2017). However, CR technology can be cultured in 3D organoids, and there are already several models that combine 3D organoid systems and stromal cells. In the future, it is necessary to establish models that integrate multiple stromal cell types into the 3D culture system with CR technology to check direct epithelial/matrix interactions (Liu et al., 2017). Another limitation is the inability to distinguish tumors from normal epithelial cells because the CR method allows normal and tumor epithelial cells to grow outward. Normal cells sometimes replicate more than tumor cells, so normal cells eventually surpass culture. However, the standard CR method has some modifications and may be a selective expansion of tumor cells *in vitro* (Wang et al., 2020). Despite these limitations, CR technology still has very good application prospects in digestive system diseases. There is no a single perfect model for biomedical research including *in vitro* or *ex vivo* cell cultures and *in vivo* animal assays since every method or system have its own unique properties, advantages and limitations/disadvantages. One chooses a model which is appropriate for his/her specific question. In many cases, scientists or researchers must choose combination of the technologies at variety of levels from molecules, cells, organs, to individual or population for their research purpose. For example, combination of CRC (2D), organoids (3D) and PDX (*in vivo*) is highly recommended from NCI (National Cancer Institute) to establish patient-derived model repository.

## Conclusion

**FIGURE 2:**
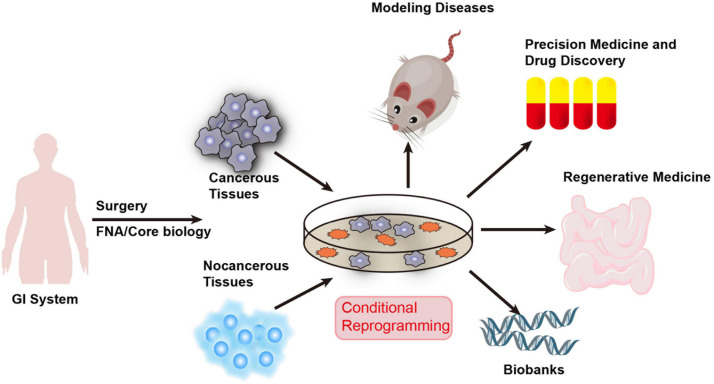
Applications of CR technology in human digestive system diseases. CR technology can quickly generate cultures from fresh and cryopreserved normal and diseased human tissue samples obtained through surgery, core biopsy, and fine-needle aspiration (FNA). Therefore, CR technology can be used as an ideal *in vitro* model for digestive system diseases and facilitate precision medicine and drug discovery. It will also aid in tissue engineering for personalized regenerative medicine and provides an exciting opportunity to build a living biobank for digestive system disease.

The development of CR technology creates exciting possibilities for digestive system research. The CR method can quickly and efficiently establish cell cultures from normal and diseased tissues. Most importantly, CRCs maintain the developmental potential of the original tissue and can restore the differentiation ability of the cells after removing these conditions. Additionally, it can quickly generate cultures from small biopsies and cryopreserved tissues. CR cells can also be produced from xenografts and organoid tissues. It can also form CDX tumors and be cultured in spheroids or organoids. Therefore, CR technology might be an ideal *in vitro* model for digestive system diseases and could facilitate precision medicine and drug discovery (Figure 2). Future studies using CR technology will also aid in tissue engineering for personalized regenerative medicine and provide an exciting opportunity to build a living biobank for digestive system disease. The use of CR technology has created great opportunities for the advancement of diagnosis, development of new treatments, and prevention of digestive system disease.

## Author Contributions

XL had the idea for the article and revised the manuscript. RZ and RL performed the literature search and drafted the manuscript. TA revised the manuscript. All authors contributed to the article and approved the submitted version.

## Conflict of Interest

Several patents for conditional reprogramming technology has been awarded to Georgetown University by the United States Patent Office. The license for this technology has been given to a Maryland-based start-up company for commercialization. The inventor, XL and Georgetown University receive potential royalties and payments from the company. Several organizations and companies (Propagenix, ATCC, STEMCELL Technologies, etc.) are selling CR cells, media and related reagents. The remaining authors declare that the research was conducted in the absence of any commercial or financial relationships that could be construed as a potential conflict of interest.
